# Syncope after aortic valve surgery

**DOI:** 10.1007/s12471-019-1243-4

**Published:** 2019-02-11

**Authors:** C. Crooijmans, L. M. Rademakers

**Affiliations:** Department of Cardiology, Catharina Ziekenhuis, Eindhoven, The Netherlands

## Answer

The electrocardiogram (ECG) shown in Fig. 2 of the rhythm puzzle question shows a wide complex tachycardia of 230 beats per minute (bpm) with a vertical axis and typical right bundle branch block. The patient underwent an electrophysiology study, which demonstrated a prolonged His-ventricular interval during sinus rhythm, indicating significant impairment of the His-Purkinje conduction system. The clinical tachycardia was easily induced by programmed stimulation. Analysis revealed participation of the His-Purkinje conduction system in the tachycardia mechanism. Thus, the patient was diagnosed with bundle branch re-entrant ventricular tachycardia (BBRVT).

BBRVT, in which both bundles are part of the re-entrant circuit, can be seen in patients with acquired heart disease and substantial conduction delay in the His-Purkinje conduction system. In this case, aortic valve surgery may also have damaged the His-Purkinje conduction system due to its close proximity to the valvular annulus. Patients usually present with presyncope or syncope due to fast ventricular rates, typically above 200 bpm. The surface ECG QRS morphology shows a characteristic (left or right) bundle branch block pattern. The diagnosis is based on findings during the electrophysiology study. Radiofrequency catheter ablation of the RBB is the first-line therapy because BBRVT has limited response to antiarrhythmic drugs [1]. In our patient ablation of the right bundle branch resulted in non-inducibility of ventricular tachycardia during programmed stimulation.
